# The impact of the COVID-19 pandemic on job satisfaction: A mediated moderation model using job stress and organizational resilience in the hotel industry of Taiwan

**DOI:** 10.1016/j.heliyon.2022.e09134

**Published:** 2022-03-19

**Authors:** Shao-Cheng Cheng, Yu-Huan Kao

**Affiliations:** a Graduate Institute of International Trade, Chinese Culture University, 55, Hwa-Kang Road, Yang-Ming-Shan, Taipei, Taiwan; b Fashion Creative Industry & Branding Management, Chinese Culture University, 55, Hwa-Kang Road, Yang-Ming-Shan, Taipei, Taiwan

**Keywords:** Perceived threat, Job stress, Job satisfaction, Organizational resilience, COVID-19, Business downturn, Hotel industry, Taiwan

## Abstract

The COVID-19 pandemic has affected businesses worldwide, including the hotel industry in Taiwan. This study aims to explore the influence of the perceived threat of COVID-19 on job satisfaction. SPSS software was used for data analysis, and the PROCESS macro was used to test the mediation, moderation, and moderated mediation hypotheses. An online survey (n = 681) was conducted on hotels in Taiwan in 2021, and the results indicate that job stress activation has a mediating effect on employees’ job satisfaction and the hotels’ business performances. Moreover, organizational resilience has a moderating effect on job stress. This study contributes theoretically to a better understanding of the factors that determine the impact of traumatic events such as a pandemic on people's mental health. This study suggests that interventions may be carried out to minimize the pandemic's negative psychological consequences. The implications of this study are also applicable to hoteliers in other affected countries.

## 1 Introduction

The COVID-19 pandemic has affected people worldwide. Besides its impact on people’s health, it has restricted people’s lives, from travel to sporting events and even education (Edelheim, 2020; Sandhu and Wolf, 2020). The restrictions on travel thus resulted in low hotel occupancy rates (Gössling et al., 2020; Jiang and Wen, 2020). The Asia-Pacific region saw a decrease of 82% in tourist arrivals in January to October 2020, the Middle East recorded a decline of 73%, while Africa saw a drop of 69% over this ten-month period (UNWTO, 2020).

Job satisfaction refers to employees’ satisfaction with work-related factors, and is deemed "subjective happiness at work" (Choudhary and Saini, 2021). Job satisfaction has been the focus of management research, as it significantly affects employees’ job performance, as well as other managers’ performance indicators, such as customer satisfaction, perceived service quality, customer loyalty, and satisfaction (O'Donoghue and Tsui, 2015). The United Nations World Tourism Organization reported that the business downturn resulted from COVID-19 differs from those in the past; hence, governments and companies need to adopt different measures in response (UNWTO, 2020).

Job stress is defined as employee’s personal subjective feelings toward the environment. When important goals and results are not achieved, the employees may feel both physical and psychological stress. Moreover, job stress is also the main reason for employees’ poor physical and mental health (Parker and DeCotiis, 1983; Haver et al., 2019). Prior research also verified that employees in the service industry have higher job stress, especially frontline employees, and job stress affects their job satisfaction (Cheng and Yang, 2018; Tongchaiprasit and Ariyabuddhiphongs, 2016).

The concept of resilience is originated from ecology, and is defined as the system that absorbs changes and can continue to develop under disturbances and changing conditions (Holling, 1973). In the field of business administration, organizations develop resilience in response to competitors’ strategy changes and emergent events (Williams and Shepherd, 2016). An organization's capabilities and strategies to face crises affect its sustainable development and increase its chance of survival after crisis (Prayag et al., 2018).

The hotel industry is the first industry under the impact of COVID-19; thus the industry’s ability to adapt to disasters and recover from emergencies is very important (Melián-Alzola et al., 2020; Romagosa, 2020). With the hotel industry facing the COVID-19 pandemic, can strong organizational resilience reduce the job stress of employees under the epidemic threat, thereby improving their job satisfaction? This question has not been answered in current research. Therefore, this study aims to explore the relationship among the perceived threat of COVID-19, organizational resilience, job stress, and job satisfaction, as well as whether the hotel employees’ feelings toward the epidemic may change under the influence of organizational resilience.

COVID-19 has triggered various psychological phenomena such as moral harm, extreme anxiety, fear of disease, depression and acute stress (Gibson and Janikova, 2021; Phillips and Kucera, 2021; Lewis and Zauskova, 2021), and the epidemic affects the work of corporate personnel insecurity, job instability and job satisfaction (Nemțeanu and Dabija, 2021; Nemțeanu et al., 2021). Therefore, this study will help to make up for the gap in the above research. The survey of hotel staff's work stress and job satisfaction also includes organizational resilience into the discussion, which is one of the contributions of this research in the academic field. Furthermore, this research can better understand the impact of the epidemic on human psychology, and expand the application of knowledge in the fields of human resource management and organizational resilience. The findings can provide managerial implications to the hotel industry.

## 2 Literature review and hypothesis development

### 2.1 The effect of the perceived threat of COVID-19 on job stress and job satisfaction

Natural disasters not only cause deaths and injuries to the people, but also change people’s living environment and daily routines, resulting in their psychological threat and undermining their subjective well-being (Bonanno et al., 2010). The Fukushima Daiichi nuclear disaster in 2013 has resulted in serious impacts on the lives and psychological well-being of the disaster victims (Murakami et al., 2020). However, scholars have seldom studied how disaster threat perceptions may affect the individuals in corporate workplaces, as well as the negative impacts on employees' organizational performance (De Clercq et al., 2017; Toker et al., 2015). After the outbreak of COVID-19, there is an increasing number of research on employees' loyalty in organizational citizenship behavior, life satisfaction, and depression (Park et al., 2021; Yan et al., 2021).

The COVID-19 pandemic has affected people’s daily life, social life, and consumption patterns (Kim, 2020). For example, people’s perception of threats under COVID-19 has increased the amount and frequency of their alcohol consumption (Rodriguez et al., 2020). Prior studies also attempt to understand whether fake news content during the COVID-19 pandemic affects people's fears (Lăzăroiu and Adams, 2020; Ljungholm and Olah, 2020; Sheares et al., 2020). People feel anxious about threat perception, particularly under the threat of COVID-19 (Krupić et al., 2020; Paredes et al., 2021). Previous literature has suggested that people’s anxiety arises from the spread of the epidemic when they do not believe that the epidemic can be controlled effectively, and they would perceive the epidemic as a threat (Goodwin et al., 2021). Prior research show that COVID-19 affects people's emotions such as extreme anxiety, fear of disease, depression and psychological stress (Gibson and Janikova, 2021; Phillips and Kucera, 2021; Lewis and Zauskova, 2021). Therefore, it is a very reasonable speculation that people's fear of disease affects their emotions and then affects employees' work psychology.

Job stress forms a negative relationship between the individual and the environment. When the individual perceives the job stress to be overwhelming, physical and mental problems may occur (He et al., 2020). French and Kahn (1962) are the first to introduce the concept of job stress into business management. Job stress is produced by the discrepancy between personal ability, external resources, and the job’s requirements (French, 1974). Parker and DeCotiis (1983) further defined job stress as an employee’s awareness or feeling of personal dysfunction as a result of perceived conditions and the employee’s psychological and physiological reactions to feeling uncomfortable, undesirable, or even threatened in the workplace. Research has shown that hospitality employees work under a highly stressful environment (Park et al., 2020).

The perception of external environment threats brings psychological pressure to employees, Scholars have suggested that people exposed to these threats may experience increased strain when performing their jobs, and the threats of external environment lead to lower job performance (Xie and Johns, 1995). The work-related anxiety caused by the perception of environmental threats to employees affects job stress and work performance (Jaramillo et al., 2006). Scholars have pointed out that the prevalent job stress in the hotel industry affects employee burnout, turnover rate, and service performance (Akgunduz, 2015; Park et al., 2020). Due to the threat of COVID-19, hotel employees feel a sense of insecurity and doubt towards their working environment and their own health. Thus, we hypothesize that when hotel employees perceive more serious threat from COVID-19, they feel greater job stress.

Stress is an unpleasant emotional experience associated with affective states, and is caused by specific events (Parker and DeCotiis, 1983). Given the nature of hospitality work, employees have to face intensive social interactions that may lead to stressful events and those events are the main antecedents of job stress (Park et al., 2020; Yang et al., 2020). COVID-19 has reduced the frequency of travels and affected hotel business performance. This is also an external factor that increases the work pressure of hotel management and employees (Jiang and Wen, 2020).

Job satisfaction is one of the most frequently studied aspects in organizational behavior research, and a topic of wide interest to both employees and company managers (Castellacci and Viñas-Bardolet, 2019; Rafferty and Griffin, 2009). Job satisfaction can also be said to be a kind of subject well-being at work (Choudhary and Saini, 2021). Subjective well-being is the main research focus in positive psychology, which includes happiness, life satisfaction, and positive emotions. Scholars have suggested that traditional research has overemphasized happiness, while neglecting positive subjective well-being (Diener, 1984; Csikszentmihalyi and Seligman, 2000).

Job stress and job satisfaction have an influential relationship. The job stress of employees will affect their work attitude, such as job satisfaction and job performance (Parker and DeCotiis, 1983; Tetrick and LaRocco, 1987). Babin and Boles (1996) used 328 front-line employees in upscale retail businesses to analyze the relationship between job stress and job satisfaction, and found that stress levels negatively relate to job satisfaction. Furthermore, Jamal and Preena (1998) indicated that job stress significantly relates to organizational commitment, overall job satisfaction, satisfaction with pay, and relations with co-workers and supervisors.

Researchers have verified the impact of personality traits on various human resource outcomes. Some researchers have applied combinations of various personality dimensions as overall individual profiles, and found that personality profiles led to varying levels of job satisfaction (Lan et al., 2021). In addition, an employee’s own service style also affects one’s job satisfaction through work engagement (Ozturk et al., 2021). Moreover, organization managers have an influence on employees. Research has found that employees’ perceptions of diversity management have a positive and direct effect on job satisfaction (García-Rodríguez et al., 2020). Past studies have suggested that work stress negatively correlates with job satisfaction (Jamal, 1990; Tetrick and LaRocco, 1987; Parker and DeCotiis, 1983). Therefore, we hypothesize that the perceived threat of COVID-19 increases job stress, which in turn, has a negative impact on job satisfaction.

Hypothesis 1 The degree of perceived threat of COVID-19 has a negative effect on job satisfaction

Hypothesis 2 The degree of perceived threat of COVID-19 has an indirect negative effect on job satisfaction mediated by its positive effect on job stress

### 2.2 The moderating role of organizational resilience

Organizational resilience is an organization’s ability to response to crisis. Through strategic adjustments and actual response actions, organizations can respond to changes smoothly (Prayag et al., 2018). Tyrrell and Johnston (2008) defined tourism resilience as the social, economic, or ecosystem capabilities that help to recover from tourism stress. Organizational resilience includes proactive organizational resilience, which is to build organizations’ readiness for changes, and reactive organizational resilience, which is to respond to and recover from crises (Jia et a., 2020). In addition, some scholars have defined resilience as leadership and management, core competence of staff, market sensitivity, situational awareness, and preparedness plans (Tibay et al., 2018).

Resilience of an enterprise during a crisis is very important for crisis management and survival of the enterprise. Organizational resilience affects the organization's adjustment of operating strategies during a crisis, and also affects employees' work experience and pressure in the organization (Tibay et al., 2018). Scholars have indicated that companies should focus on building complete resilience of mobility, rather than building step-by-step planning capability (Somers, 2009). A team demonstrates its organizational resilience in an uncertain environment by adjusting its strategies, cooperating with its strategic partners, employee participation, continuous innovation and learning, and changes in management as the environment changes (Melián-Alzola et al., 2020).

In previous studies on organizational resilience, scholars have found a significantly positive relationship between employee resilience and mental state in the tourism industry. Employee resilience can help to increase the organizational resilience of the tourism industry, and employee resilience can also increase life satisfaction (Prayag et al., 2018). Scholars have argued that trust, which is composed of competence, openness, and reliability, has a strengthening effect on organizational resilience (Olu-Daniels and Nwibere, 2014). Resilient leadership explains the role of variables related to the development of organizational resilience, which is affected by the adaptation capacity of the organizations through the factors of the organizational culture and the abilities to organize and manage operations (Morales et al., 2019). Organizations can affect resilience factors, among which organizational leadership and management are the most influential factors on organizational resilience, and organizational resilience affects organizational operational performance and employee work status (Tibay et al., 2018). Organizational resilience can also reduce work pressure, and the reduction of job stress is critical to reducing turnover (Lee et al., 2020). We hypothesize that job stress mediates the indirect effect of the perceived threat of COVID-19 on job satisfaction, and when the resilience is low, the mediating effect is higher.

Hypothesis 3 The indirect effect of the degree of perceived threat of COVID-19 on job satisfaction through activating job stress is moderated negatively by the organization’s degree of resilient.

**Figure 1:**
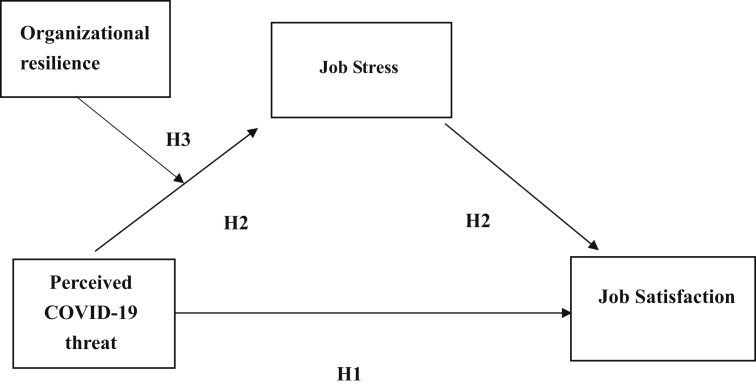
The Conceptual Framework. H = hypothesis.

Based on these three hypotheses, the conceptual model proposed is shown in Figure 1.

## 3 Method

### 3.1 Participants and procedure

The hotel industry has faced many significant challenges, and the COVID-19 pandemic has had a distinctly destructive economic impact around the world (Goodell, 2020). The impact on hospitality and travel in particular has been severe (Nicola et al., 2020). Governments and companies must be more cautious in facing this pandemic (UNWTO, 2020). In the hotel industry that is impacted by COVID-19, employees working in the international tourist hotels are required to meet more diverse customer expectations than those working in local hotels (Teng, 2019). Therefore, this study targeted frontline employees in international tourist hotels in Taiwan. However, this study excluded employees with less than six months of service in the hotel, considering their insufficient experience working in the hotel (Sias, 2005). The title of ethical approval committee's and respondents' consent for using survey to collect data.

The study was conducted from May to August 2021 during severe COVID-19 lockdown measures in Taiwan. To ensure the validity of the questionnaire, 30 pre-tests were completed. The research team distributed the questionnaires, gave respondents a cover letter explaining the research purposes, and assured them that the results would remain confidential. To reduce common method variance (CMV), temporal separation was conducted (Podsakoff et al., 2012).

### 3.2 Measures

The original questionnaire was in English and was then translated into Chinese. To ensure translation accuracy, five experts in the hotel industry were invited to review the questionnaire. The measurement was based on a 7-point Likert-type scale ranging from 1 (strongly disagree) to 7 (strongly agree). We received at least 50 questionnaires from each hotel (A total of 15 hotels). After eliminating 69 invalid questionnaires containing conflicting, we collected 681 useful questionnaires, yielding a response rate of 90%. Type of the study is Cross-sectional. We conducted a pilot test on a sample of 30 full-time hotel employees in Taiwan to ensure the clarity, reliability, and comprehensiveness of the questionnaire. We reworded some of the questionnaire items based on the pilot test.

The analyzed variables were assessed with validated measurement scales stemming from the literature (see Table 1). The measure for perceived threat, which contains 3 items, was adopted from Rodriguezet al. (2020). In this study, the scale was internally consistent and had a Cronbach's alpha coefficient of 0.794. Next, the measure for job satisfaction, which contains 7 items, was adopted from Seemaet al., (2021). In this study, the scale was internally consistent and had a Cronbach's alpha coefficient of 0.947. To measure job stress, the 4-item scale was adopted from Schwepker and Dimitriou (2021), which was modified from House, & Rizzo (1972). Responses were defined job stress as an individual’s subjective feeling towards the surrounding environment. Cronbach’s alpha for this scale was 0.753, computed by averaging the reliability of this scale across 10 days. The organizational resilience scale, containing 3 items, was adapted from Melián-Alzolaet al. (2020). In this study, the scale was internally consistent and had a Cronbach's alpha coefficient of 0.906.

**Table 1:** Variables and measurement items.

Variables and Dimensions	Mean	Standard Deviation	Factor Loading	(CR)	(AVE)
Perceived Threat (Rodriguez et al., 2020)	PT1. Thinking about the coronavirus (COVID-19) makes me feel threatened.	.048	.718	0.672	0.693
	PT2. I am afraid of the coronavirus (COVID-19)	.045	.902		
	PT3. I am stressed around other people because I worry I’ll catch the coronavirus.	.048	.900		
Job Satisfaction (Choudhary and Saini, 2021)	JSA1. My Job is usually interesting enough to keep me away from getting bored	.041	.932	0.724	0.745
	JSA 2. I enjoy my work more than my leisure time	.045	.902		
	JSA 3. I feel fairly satisfied with my job	.041	.846		
	JSA 4. I feel that I am happier in my work than most other people	.041	.781		
	JSA 5. Most days I am enthusiastic about my work	.044	.807		
	JSA 6. I like my job better than the average worker does	.042	.929		
	JSA 7. I feel real enjoyment in my work	.046	.895		
Job Stress (Schwepker and Dimitriou, 2021)	JS1. I feel fidgety or nervous because of my job.	.052	.890	0.692	0.704
	JS2. Problems associated with work have kept me awake at night.	.054	.884		
	JS3. I feel nervous before attending meetings in this organization	.059	.585		
	JS4. If I had a different job, my health would probably improve.	.042	.524		
Organizational Resilient (Melián-Alzola et al., 2020)	OR1. Achieves a new organizational balance by adapting to changes in the environment (offering new products or services, incorporating new technologies, negotiating with tour operators...)	.050	.921	0.665	0.724
	OR2. Recovers and strengthens at a strategic and operational level (recovering the hotel occupancy rate, improving its competitive position...)	.050	.922		
	OR3. Adapts strategically and operationally to new environmental conditions	.047	.906		

The variables are in italics. The measurement items in regular text. The variables are computed by averaging the corresponding measurement items.

## 4 Data analysis and results

### 4.1 Data analysis

A total of 681 Chinese adults (54.6% female and 45.4% male) participated in the current study. In addition, participants completed a questionnaire on basic socio-demographic data. Detailed characteristics of the group in terms of education, age and job occupation are presented in Table 2.

**Table 2:** Sample characteristics.

Characteristic	N = 681	%
**Gender**		
Male	309	45.4
Female	372	54.6
**Age (years)**		
21–30	137	20.1
31–40	296	43.5
41–50	221	32.5
50 and over	27	4
**Level of education**		
Secondary education	173	25.4
University education	483	70.9
Master’s or PhD	25	3.7

SPSS 25.0 and SPSS PROCESS macro 3.4 software (Hayes and Preacher, 2013; Hayes, 2017) were used to analyze the data. PROCESS macro was used to test mediation, moderation, and the moderated mediation hypotheses. All regression coefficients were tested by the bias-corrected percentile Bootstrap method. The theoretical model was tested by estimating the 95% confidence interval (CI) for mediation and moderating effects with 5,000 sampled with repetition. ​To answer the question concerning moderation, we added organizational resilience to the model as a potential moderator for both the direct effect and the path between perceived threat and job satisfaction (moderated mediation; PROCESS Model 7).

### 4.2 Results

A correlation analysis (Table 3) confirmed a significant positive relationship between the perceived threat of the COVID-19 pandemic and job stress (r = 0.345, p < .01) and a significant negative relationship between job stress and job satisfaction (r = -0.352, p < .01). The results also confirm a significant negative relationship between the perceived threat of COVID-19 and job satisfaction (r = -0.623, p < .01), which supports H1.

**Table 3:** Variable correlations.

	PT	JS	JSA	OR
Perceived Threat (PT)	1			
Job Stress (JS)	.345∗∗	1		
Job Satisfaction (JSA)	-.623∗∗	-.352∗∗	1	
Organizational Resilient (OR)	-.352∗∗	-.452∗∗	.451∗∗	1

∗∗p < .01.

Mediation analysis of indirect effects using Hayes' (2017) PROCESS with 10,000 bootstrap samples and bias-corrected bootstrap confidence intervals (Hayes, 2017) confirmed that the effect of the perceived COVID-19 threat on job satisfaction was mediated by job stress (b_ind_ = 0.345, Boot SE = 0.045, 95% Boot CI [−0.352, -0.134]), which supports H2.

Moderated mediation analysis using PROCESS on the moderating effect of organizational resilience found a significant moderated mediation index (10,000 bootstrap samples; b_modmed_ = -0.244, SE = 0.0024, 95% Boot CI [−0.324, -0.134]).

**Figure 2:**
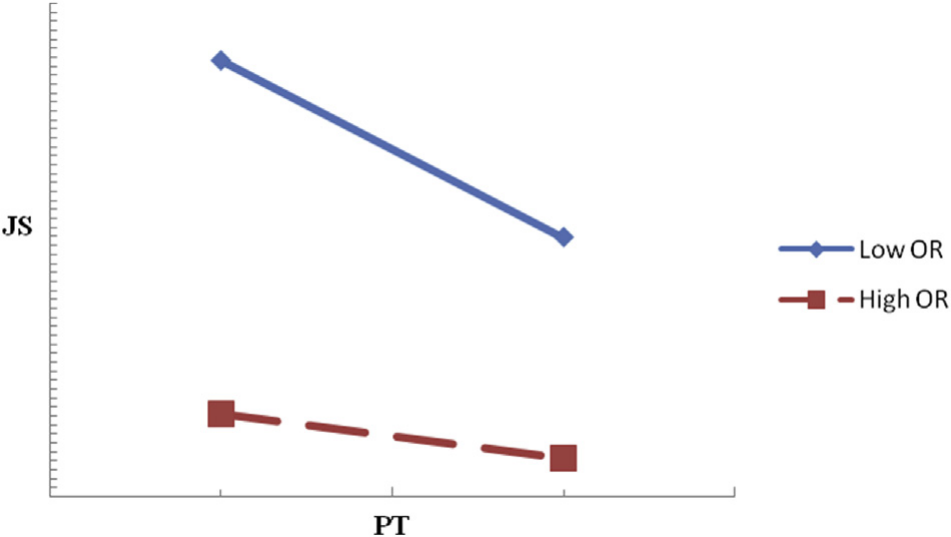
Effect of low and high perceived threat of the COVID-19 pandemic on job satisfaction mediated by job stress, at high and low values of moderator organizational resilience.

Therefore, the results confirm that the indirect effect of the perceived threat of the pandemic on job satisfaction through job stress was moderated by the level of organizational resilience, providing support for H3. Table 4 and Figure 2 present the pattern of moderation at different values of the moderator organizational resilience.

**Table 4:** Moderated mediation analysis of indirect effect of the perceived threat of COVID-19 on job satisfaction through activating job stress.

Index of moderated mediation						
Mod.	Med.	Mod. Med. Index		Boot SE	Boot LLCI	Boot ULCI
OR	JS	-0.244		0.0024	-0.324	-0.134

**Conditional indirect effect at values of the moderator**						
**Mod.**	**Med.**	**Values Mod.**	**Cond Index effect**	**Boot SE**	**Boot LLCI**	**Boot ULCI**
OR	JS	5.280	-0.352	0.024	-0.456	-0.224
		4.674	0.345	0.045	0.252	0.462
		4.966	-0.134	0.035	-0.245	-0.104

Note. 10,000 bootstrap samples for bias-corrected 95% bootstrap confidence intervals, Boot SE = Bootstrap standard error, Boot LLCI = Bootstrap lower limit confidence interval, Boot ULCI = Bootstrap upper limit confidence interval, values for quantitative moderators are the mean (M) and plus/minus one SD from mean (−1SD/+1SD), Mod.: moderator, Med.: mediator, OR: Organizational Resilient, JS: Job Stress.

## 5 Conclusion

This study explored the influence of the perceived threat of COVID-19 on job satisfaction. The survey results support all three hypotheses. The contributions and implications of this study are detailed.

### 5.1 Theoretical contributions

This study examined the relationship between the perceived threat of COVID-19 and job satisfaction in the hotel industry, specifically considering the mediating role of job stress and the moderating role of organizational resilience. The results of this study support the first two proposed hypotheses, indicating that the perceived threat of COVID-19 is negatively correlated with job satisfaction (H1), and that job stress mediates the association between the perceived threat of COVID-19 and job satisfaction (H2). The results also show that organizational resilience moderates the relationship between the perceived threat of COVID-19 and job stress (H3).

Prior studies show that COVID-19 has triggered various psychological phenomena such as moral harm, extreme anxiety, fear of disease, depression and acute stress (Gibson and Janikova, 2021; Phillips and Kucera, 2021; Lewis and Zauskova, 2021). The results of this study found that corporate employees' perceived COVID-19 threat would indeed lead to an increase in work stress, which is the empirical contribution of this study to the psychological phenomenon of the epidemic affecting people. Furthermore, COVID-19 affects the work of corporate personnel insecurity, job instability and job satisfaction (Nemțeanu and Dabija, 2021). The findings of this study also validate the relationship between job stress and job satisfaction. Those above are the substantial contributions of this research to the academic field.

COVID-19 has changed people’s lifestyle and health, and in turn, affecting people’s mental health and emotions (Bonanno et al., 2010; Murakami et al., 2020; Park et al., 2021). Previous studies have considered the influence of different institutional and organizational factors, and proposed different models of working conditions for European hospitality workers that result in different levels of job satisfaction (Díaz-Carriónb et al., 2020). This study found that the perceived threat of COVID-19 is negatively associated with job satisfaction, which can prove that the disaster has a considerable impact on the job satisfaction of employees. This finding is consistent with previous studies on the impact of disasters on people’s psychological well-being. The number of guests and revenue of the hotel industry have reduced because of the COVID-19 and have decreased employees’ job satisfaction. This finding also echoes with previous studies on the organizational citizenship behavior, life satisfaction, and depression of corporate employees under the impact of COVID-19 (Park et al., 2021; Yan et al., 2021).

The research contribution of this study is to verify the impact of disaster threats, well as to explore the relationship between threat perception, job stress, and job satisfaction, and the mediating effect of job stress. People feel anxious about the future due to their threat perception, and they feel stronger anxiety if their perceived epidemic controllability is low (Goodwin et al., 2021; Krupić et al., 2020; Paredes et al., 2021). The work-related anxiety caused by the perception of environmental threats on employees can affect work stress (Jaramillo et al., 2006). The results of this study confirm that COVID-19 increases the job stress of employees, which in turn affects their job satisfaction. As previous studies on COVID-19 did not simultaneously investigate the relationship between threat perception, job stress, and job satisfaction, the results of this study can make up for this research gap and provide further insights into this topic. Employees in the service industry generally perceive high work pressure, which affects their burnout, turnover rate, and service performance (Akgunduz, 2015; Park et al., 2020). This study notes that even awareness of the threat of an epidemic affects job stress and job satisfaction. Therefore, future studies can apply the results of this study to expand the exploration of the impact of customer satisfaction and employee turnover intentions.

Organizational resilience affects an organization's adjustment of its operating strategies in a crisis and also affects employees' work experience and pressure in the organization (Tibay et al., 2018). This study found that organizational resilience has a moderating effect on the perceived threat of COVID-19 and job stress. The results support the research hypotheses. When a team’s continuous adjustment strategy and management also change with environmental changes, its organizational resilience is stronger in an uncertain environment (Melián-Alzola et al., 2020). Employees have observed changes in the organization’s strategies and management approaches in the face of COVID-19, and judge organizational resilience, thus affecting job stress perceptions. In other words, when the organization becomes more resilient, fewer employees would counter job stress as a result of the epidemic. This also means that organizational resilience is not only a power tactic for the organization to face unfavorable external environments, but also has a positive impact on the psychological level of employees within the company.

This study discussed the impact of the COVID-19 pandemic on job satisfaction. The results indicate that job stress and job satisfaction are negatively correlated. In other words, higher job stress leads to lower job satisfaction, which confirms the findings of previous studies that when employees feel stressed at work, then this will influence their work attitude such as job satisfaction (Babin and Boles, 1996; Flanagan and Flanagan, 2002). In addition, this study adopted job stress as a mediator factor to test the relationship between perceived threat and job satisfaction. The results suggest that perceived threat and job satisfaction have a significantly negative correlation, which means that any perceived threat would decrease job satisfaction.

Finally yet importantly, this study found that organizational resilience has a moderating effect on the relationship between perceived threat and job stress. This result is consistent with prior studies in which organizational resilience affects organizational operational performance and employee work status (Tibay et al., 2018).

### 5.2 Practical implications

#### 5.2.1 Job stress management

Employee face great work pressure in a highly competitive work environment. Although appropriate pressure can stimulate employees to work harder, excessive job pressure that cannot be relieved properly would lead to adverse physical and mental effects, even affecting the organizational performances. The perception of external environmental threats can bring about psychological pressure on employees (Xie and Johns, 1995). Pressure in the service industry is inherently higher than that in the general industry, and the job stress of employees is greater (Cheng and Yang, 2018; Tongchaiprasit and Ariyabuddhiphongs, 2016). Therefore, when epidemic threats result in greater job stress for the employees, business managers must pay attention to the job stress faced by the employees and resolve them in a timely manner, so as to maintain high morale of employees and provide good service quality.

#### 5.2.2 Demonstration of organizational resilience

Organizational resilience affects an organization's operational performance, and reduces work pressure. Reducing job stress is extremely important to reducing turnover (Lee et al., 2020; Tibay et al., 2018). This study verified that organizational resilience has a moderating effect between threat perception and job stress. When a company faces an externally unfavorable business environment, organizational resilience has a positive impact on employee psychology. Therefore, business managers should promote resilience to effectively respond to changes in the environment, and more importantly, enhance the employees' morale. During an epidemic, companies can reduce the job stress of employees, thereby increasing job satisfaction and reducing turnover.

#### 5.2.3 Recruit and retain highly resilient employees

COVID-19 has brought operating difficulties and challenges to many industries around the world, and companies must have the ability to adapt to disasters and recover from emergencies (Melián-Alzola et al., 2020; Romagosa, 2020). Previous research has found a significantly positive relationship between employee resilience and psychology, and employee resilience also helps to increase organizational resilience (Chowdhury et al., 2020). Therefore, in order to effectively overcome the business problems caused by COVID-19, companies could recruit new employees with high resilience by using a resilience test. In situations that companies need to lay off employees, employees should be retained as much as possible, so as to improve the overall organizational resilience.

### 5.3 Limitations and future research

This study has some limitations. First, the scope of research was within Taiwan, and the sampled population was only hotel employees. Thus, the results of this study cannot be generalized to employees of other industries or other regions. Future studies can expand the scope to other regions of the world and across different industries. Second, this cross-sectional study did not make causal inferences between the variables. However, the research variables of this study were selected from the existing literature, and reasonably deduced and verified; this is another limitation of this study. Third, this study did not consider the demographic variables of employees, such as gender, age, working experience, etc. As previous studies have found that personality traits are related to job satisfaction (Lan et al., 2021), the findings of this study are limited to the job stress and job satisfaction of hotel employees in Taiwan during the COVID-19 pandemic period.

Future research direction can be towards the following.

Future research can add other variables, such as work engagement, burnout, and service performance to discuss the impact of the perceived threat of COVID-19. Second, as the work pressure of hotel employees is generally high (Park et al., 2020), researchers can conduct longitudinal studies to track changes in employees’ job stress before and after the COVID-19 period, in order to better understand the effect of the perceived threat of COVID-19 on job stress.

Third, through the factors of the organizational culture, as well as the abilities to organize and manage operations, the adaptation capacity of the organizations affects the development of organizational resilience (Morales et al., 2019). This study only discussed the impact of organizational resilience on job stress; hence, future research can further explore the component variables of organizational resilience, as well as establish a complete model for the impact of external environmental threats on the organization.

Fourth, this study investigated the psychological state of employees under the perceived threat of COVID-19. Future studies can survey the hotel guests on the service quality of hotel employees during the pandemic period for comparison. In this way, researchers can understand if the perceived threat of COVID-19 affects employees' work pressure and job satisfaction, and whether this possibly reduces service quality. The findings herein would be more complete if hotel guests’ opinions can also be included.

Finally, Previous studies have focused on the psychological impact of COVID-19 misinformation and fake news (Lăzăroiu and Adams, 2020; Ljungholm and Olah, 2020). However, this study did not explore the influence of the authenticity of the source of information on people's fear, and follow-up research can also explore in this direction.

## Declarations

### Author contribution statement

Shao-Cheng Cheng: Conceived and designed the experiments; Contributed reagents, materials, analysis tools or data; Wrote the paper.

Yu-Huan Kao: Performed the experiments; Analyzed and interpreted the data; Wrote the paper.

### Funding statement

This research did not receive any specific grant from funding agencies in the public, commercial, or not-for-profit sectors.

### Data availability statement

Data included in article/supplementary material/referenced in article.

### Declaration of interests statement

The authors declare no conflict of interest.

### Additional information

No additional information is available for this paper.
